# Regulation of aquaporin‐4 expression in the central nervous system investigated using M23‐AQP4 null mouse

**DOI:** 10.1002/glia.24032

**Published:** 2021-05-26

**Authors:** Francesco Pisani, Laura Simone, Maria Grazia Mola, Manuela De Bellis, Antonio Frigeri, Grazia Paola Nicchia, Maria Svelto

**Affiliations:** ^1^ Department of Biosciences, Biotechnologies and Biopharmaceutics University of Bari Aldo Moro Bari Italy; ^2^ Fondazione IRCCS Casa Sollievo della Sofferenza, Cancer Stem Cells Unit San Giovanni Rotondo (FG) Italy; ^3^ School of Medicine, Department of Basic Medical Sciences, Neuroscience and Sense Organs University of Bari Aldo Moro Bari Italy; ^4^ Institute of Biomembranes and Bioenergetics National Research Council Bari Italy; ^5^ National Institute of Biostructures and Biosystems (INBB) Rome Italy

**Keywords:** AQP4 mRNA binding proteins, CRISPR/Cas9 M23‐null mouse model, DDX17, PTBP1, translational regulation

## Abstract

In astrocytes, unknown mechanisms regulate the expression of M1 and M23 isoforms of water channel aquaporin‐4 (M1‐AQP4 and M23‐AQP4). The ratio between these two isoforms controls the AQP4 assembly state in the plasma membrane known as orthogonal arrays of particles (OAPs). To give new insights into these mechanisms, here, we explore the regulation of AQP4 expression in the spinal cord of a CRISPR/Cas9 M23‐null mouse model (M23‐null). In the M23‐null spinal cord OAP assembly, the perivascular localization of AQP4 and M1‐AQP4 protein were drastically reduced. In heterozygous, M1‐AQP4 was proportionally reduced with M23‐AQP4, maintaining the isoform ratio unaffected. We hypothesize a role of the M23‐AQP4 in the regulation of M1‐AQP4 expression. M1‐AQP4 transcription, splicing and M1‐AQP4 protein degradation were found to be unaffected in M23‐null spinal cord and in M23‐null astrocyte primary culture. The translational control was investigated by mRNA‐protein pull down and quantitative mass spectrometry, to isolate and quantify AQP4 mRNA binding proteins (AQP4‐RBPs). Compared to WT, in M23‐null spinal cord, the interaction between AQP4 mRNA and polypyrimidine tract binding protein 1, a positive regulator of AQP4 translation, was higher, while interaction with the RNA helicase DDX17 was lower. In astrocyte primary cultures, DDX17 knockdown upregulated AQP4 protein expression and increased cell swelling, leaving AQP4 mRNA levels unchanged. Here, we identify AQP4‐RBPs and provide evidence that in mouse spinal cord M23‐AQP4 deletion changes the interaction between AQP4 mRNA and some RBPs involved in AQP4 translation. We describe for the first time the RNA helicase DDX17 as a regulator of AQP4 expression in astrocytes.

## Main Points

- Aquaporin‐4 (AQP4) is regulated by translational mechanism in the central nervous system.
- AQP4‐M23 deletion changes the interaction between DDX17 and PTBP1 with AQP4 mRNA.
- DDX17 and PTBP1 regulate AQP4 expression in astrocytes.

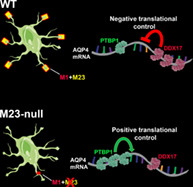

## 1 INTRODUCTION

The glial water channel aquaporin‐4 (AQP4) is expressed as two main isoforms M1‐AQP4 (32 kDa) and M23‐AQP4 (30 kDa) originating by alternative translational initiation mechanism (Rossi et al., 2010). The ratio between these two isoforms in turn controls the AQP4 supramolecular assembly state in the plasma membrane known as orthogonal arrays of particles (OAP) (Smith & Verkman, 2015).

Other isoforms, named M1‐AQP4ex and M23‐AQP4ex, have recently been characterized as being able to modulate OAPs assembly and the astrocytic endfeet localization of AQP4 (De Bellis et al., 2017; Palazzo et al., 2019; Palazzo et al., 2020). A large number of environmental, metabolic and hormonal stimuli have been reported to be able to change AQP4 expression, thereby regulating its role in physiological and pathological processes. In most of these studies, the authors have described the phenomena but have not provided any direct evidence concerning the molecular dynamics underlying AQP4 downregulation or upregulation (Costa et al., 2019; Li et al., 2019; Lichter‐Konecki et al., 2008; Murillo‐Carretero et al., 1999; Szpilbarg et al., 2018; Wang et al., 2018). Consequently, AQP4 regulation remains largely an unaddressed issue. This represents one of the main obstacles to developing a new strategy to fine‐tune AQP4‐mediated processes in which pathological AQP4 dysfunction plays an active and well documented role such as ischemia (Vella et al., 2015), brain edema (Clement et al., 2020), or neuromyelitis optica (Nicchia et al., 2009; Pisani, Mastrototaro, et al., 2011).

Some molecular dynamics related to the transcriptional regulation of AQP4 and to the microRNA‐dependent AQP4 mRNA stability have been clarified (Abe et al., 2012; Kapoor et al., 2013; Sepramaniam et al., 2012; Yi et al., 2013; Zheng et al., 2017), as well as some translational regulation mechanisms such as leaky scanning (Rossi et al., 2010), reinitiation (Pisani, Rossi, et al., 2011), read‐through (De Bellis et al., 2017), and IRES‐dependent translation (Baird et al., 2007). Most of these mechanisms were explored in vitro in transfected systems without providing direct evidence in AQP4‐expressing tissues. Among these mechanisms, only the role of the AQP4 translational read‐through in the expression of AQP4ex has recently been confirmed in human, rat, and in mouse brain by in vivo study (Palazzo et al., 2019; Palazzo et al., 2020; Sapkota et al., 2019). Whether translational regulation of AQP4 expression plays an active role in the control of AQP4 expression levels in the central nervous system (CNS) and what molecular players are involved, remains still largely unknown.

We have recently developed a CRISPR/Cas9 mouse model in which the codon of the methionine in Position 23 (M23) was replaced with the isoleucin (I) codon (Rossi et al., 2010), eliminating the synthesis of OAP‐forming M23‐AQP4 isoforms (M23‐null). We have reported that in M23‐null mice OAPs are absent, the tetrameric form of AQP4 is upregulated and the M1‐AQP4 protein expression is strongly downregulated. This demonstrates that M23‐AQP4 is pivotal to preserve the normal AQP4 expression level in the CNS (de Bellis et al., 2020). In the present work, we have analyzed the same animal model with the aim of exploring the mechanism by which M23‐AQP4 deletion controls AQP4 expression. The spinal cords of WT, M23‐null, and heterozygous mice were used in this study. AQP4 protein localization, expression, and supramolecular assembly were analyzed by confocal microscopy and biochemical procedures. The AQP4 expression was investigated at the transcriptional, splicing, translational and posttranslational levels.

The main findings come from the analysis of the translational regulation. Here, we isolate AQP4 mRNA binding proteins (RBPs) and demonstrate that M23‐AQP4 deletion changes the interaction between RBPs and AQP4 mRNA. Between these RBPs, we validate DDX17 in astrocyte primary culture as a new regulator of AQP4 expression.

The role of RBPs in the regulation of AQP4 expression has never been described before. This opens up a new perspective to clarify AQP4 regulation in physiological and pathological conditions.

## 2 MATERIALS AND METHODS

### 2.1 Ethics statement

In this study, no experiments were performed on live animals. Experiments were performed according to the European directive on animal use for research and the Italian law on animal care. The protocols were approved by the Italian Ministry of Health (Protocol No. 710/2017‐PR and 571/2018‐PR). All experiments were designed to minimize the number of animals used and animal suffering. Mice were maintained under a 12 h dark to light cycle, at constant room temperature (RT) and humidity, with food and water provided ad libitum, and supplied with environmental. enrichment materials such as toys and shelters.

### 2.2 Animals

The animal model used here was already published (de Bellis et al., 2020). OAP‐null mice harboring the M23I point mutation were generated on the C57BL/6J background by Cyagen Biosciences (Santa Clara, CA) using CRISPR/Cas9‐based targeting and homology‐directed repair. AQP4 knockout (KO) mice with a CD1 genetic background and age‐matched CD1 mice, used as WT mice, were kindly provided by Dr Hu (Nanjing Medical University, China). Genotyping was performed on tail DNA using standard protocols.

### 2.3 Antibodies

The primary antibodies used were: goat polyclonal anti‐AQP4 (C‐19) (sc‐9888) and rabbit polyclonal anti‐AQP4 (H‐80) (sc‐20812) (Santa Cruz Biotechnology) both diluted 1:400 for immunofluorescence and 1:500 for immunoblot analysis; rat monoclonal anti‐CD31 at 1:100 (BD Bioscience, 550274) for immunofluorescence (GFAP); mouse monoclonal anti‐DDX17 (C‐9) (Santa Cruz Biotechnology, sc‐271112) diluted 1:100 for immunofluorescence and immunoblot analysis; rabbit anti‐DDX17 (Sigma Aldrich, SAB2100550) 1:500 in immunoblotting, mouse anti‐GAPDH (Merck Millipore, MAB374) at 1:2000; rabbit polyclonal anti‐actin (Sigma Aldrich, A2066) at 1:500; Goat anti‐polypyrimidine tract binding protein 1 (PTBP1) (Sigma Aldrich, SAB2500834) at 1:1000. An affinity‐purified rabbit polyclonal antibody to a M1‐AQP4 specific sequence was prepared by Gen‐Script and diluted 1:1000 for immunoblot analysis (de Bellis et al., 2020). The following secondary antibodies were used for Western blot diluted to 1:5000: donkey anti‐goat IgG‐horse‐radish peroxidase (HRP); goat anti‐rabbit IgG‐HRP (Santa Cruz Biotechnology) and goat anti‐mouse IgG‐HRP (Bio‐Rad). The secondary antibodies used for immunofluorescence were: donkey anti‐mouse Alexa Fluor 594‐conjugated (A21203), donkey anti‐rabbit Alexa Fluor 488–conjugated (A21207), donkey anti‐goat Alexa Fluor 488‐conjugated (A11055), and donkey anti‐rat Alexa Fluor 594 conjugated (A21203) (all from Thermo).

### 2.4 Constructs

Human and rat M1‐AQP4 and M23‐AQP4 coding sequences was cloned into pTarget (www.Promega.com). The previously characterized mutated form of M1‐AQP4 (M23I), demonstrated to give rise exclusively to AQP4‐tetramers (Rossi et al., 2010), was used.

### 2.5 Immunofluorescence and confocal microscopy

Spinal cord was isolated, fixed for 4 h in 4% PFA solution, washed in PBS, cryoprotected in 30% sucrose in PBS overnight, frozen at −80°C and sectioned in 10 μm thickness slices with a cryostat (CM 1900; Leica) at −20°C. After blocking in 3% BSA and 0.3% TRITON X‐100 in PBS solution, sections were incubated with primary antibodies overnight at 4°C in blocking solution, washed for 30 min, incubated with secondary antibodies for 1 h in 3% BSA solution in PBS and finally, washed for 15 min in PBS and mounted with Mowiol or in PBS‐glycerol (1:1) pH 8.0, containing 1% 1,4‐diazabicyclo[2.2.2]octane (DABCO).

Astrocytes were fixed in 4% paraformaldehyde for 15 min, washed three times in PBS, and permeabilized with 0.3% Triton X‐100. After blocking using 1% BSA for 30 min at RT, cells were incubated for 1 h with primary antibodies and washed with 1% BSA PBS. Cells were finally incubated with Alexa Fluor‐conjugated secondary antibodies and washed for 15 min in PBS. Cells and tissues were mounted with Mowiol or in PBS‐glycerol (1:1) pH 8.0, containing 1% DABCO. Immunostained tissues were observed with a Leica DM6000B microscope equipped with HC PL Fluotar ×10, ×40 objectives. In all experiments, no‐primary and no‐secondary antibody controls were run in parallel. No specific staining was observed in these controls. Confocal images were obtained with Leica TCS SP5 and SP8 confocal microscopes. All confocal images were collected using 594 and 488 nm lasers for excitation and a pinhole diameter of 1 Airy unit.

### 2.6 Astrocytes primary cell culture, RNA interference, and cycloheximide chase assay

Mouse astrocyte primary cultures were prepared from newborn pups as previously described (Nicchia et al., 2000). Cells were cultured in DMEM medium supplemented with 10% fetal bovine serum (FBS), 100 U ml^−1^ penicillin and 100 mg ml^−1^ streptomycin, and maintained at 37°C in a 5% CO_2_ incubator. All reagents were purchase from Euroclone. RNA interference experiments were performed as follows: P1 astrocytes were transfected with 30 pmol of DDX17 and PTB siRNA or scrambled siRNA by Lipofectamine 3000 (ThermoFisher) in a 12 multiwell format, according to the instruction manual in high glucose DMEM‐Glutamax and analyzed after 5 days. Three independent astrocyte preparations were used. The short interfering RNAs used were: DDX17 silence selected predesigned siRNA (ThermoFisher, ID s2344296), PTB siRNA (sense sequence: UGCACCUCUCCAACAUCCCGCUU; antisense sequence: GCGGGAUGUUGGAGAGGUGCAUU) and a scrambled (target sequence: NNUGGAGAAGGCCAACUAGGG; sense: UGGAGAAGGCCAACUAGGGUU; antisense: CCCUAGUUGGCCUUCUCCAUU) (ThermoFisher). WT and M23‐null astrocyte were treated with 30 μM cycloheximide (CHX; Sigma‐Aldrich, Milan, Italy), harvested after 0, 4, and 8 h and analyzed by Western blotting.

### 2.7 Cell line and transfection

The HEK293 cell line, derived from human embryonic kidney (ATCC CRL‐1573), was grown in DMEM medium supplemented with 10% heat‐inactivated FBS, 100 UI ml^−1^ penicillin, and 100 mg ml^−1^ streptomycin, and maintained at 37°C in a 5% CO_2_ incubator.

Twenty‐four hours before transfection, the cells at 70% confluence were plated using antibiotic‐free medium. Transient transfection was carried out using Lipofectamine 2000 (Invitrogen, Milan, Italy, www.thermofisher.com) in OptiMEM growth medium according to the manufacturer's protocol and analyzed after 36 h. For protein stability assay, 36 h posttransfection, HEK293 cells were treated with 30 μM CHX (Sigma‐Aldrich) or dimethyl sulfoxide then harvested for protein extraction and evaluation of AQP4 expression levels after CHX treatment.

### 2.8 SDS‐PAGE

Isolated mice spinal cords were immediately frozen in liquid nitrogen, pulverized, and weighed. For the extraction of different animal samples, the same weight of pulverized sample was dissolved in 10 volumes of Laemmli sample buffer (Bio‐Rad), 50 mM dithiothreitol and Protease Inhibitor Cocktail (Roche), heated to 37°C for 10 min, resolved in a 13% polyacrylamide gel, and transferred onto polyvinylidene difluoride (PVDF) membranes (Immobilon PVDF; Millipore) for immunoblot analysis. Astrocyte protein extraction was carried out using TRIzol reagent (Invitrogen) according to the instruction manual, dissolved in 1%SDS and centrifuged at 10,000*g* for 10 min. The supernatants were collected, and the total protein content was measured using absorbance at 280 nm. This method was chosen to analyze the mRNA and protein of the same sample.

Transfected cells were dissolved in Laemmli sample buffer (Bio‐Rad) or in RIPA lysis buffer (10 mM Tris–HCl, pH 7.4, 140 mM NaCl, 1% Triton X‐100, 1% Na deoxycholate, 0.1% SDS, 1 mM Na_3_VO_4_, 1 mM NaF, and 1 mM EDTA). When extracted in RIPA buffer total protein content was measured by MicroBCA assay (ThermoFisher).

### 2.9 Tissues sample preparation for two‐dimensional BN‐SDS/PAGE

Isolated mice spinal cords were dissolved in seven volumes of BN buffer (1% Triton X‐100, 12 mM NaCl, 500 mM 6‐aminohexanoic acid, 20 mM Bis‐Tris, pH 7.0, 2 mM EDTA, 10% glycerol) plus Protease Inhibitor Cocktail (Roche). The tissues were lysed on ice for 1 h, and centrifuged at 21,000*g* for 30 min at 4°C. The supernatants were collected, and the total protein content was calculated using the BCA Protein Assay Kit (ThermoFisher).

### 2.10 First dimension: Blue native‐PAGE

Here, 50 μg of protein sample were mixed with 5% CBB G‐250 (Coomassie blue G‐250) and loaded onto a polyacrylamide native gradient gel (3–9%) (20153404). The running buffers were as follows: anode buffer (25 mM imidazole, pH 7) and blue cathode buffer (50 mM tricine; 7.5 mM imidazole; 0.02% Coomassie blue G‐250; pH 7). Electrophoresis was performed at 6 mA and stopped when the tracking line of the CCB G‐250 dye had left the edge of the gel. Proteins were blotted onto PVDF membranes (Millipore) for immunoblot analysis or alternatively the lanes were used for the second dimension.

### 2.11 Second dimension: SDS‐PAGE

For the 2D BN/SDS‐PAGE analysis, lanes from the first dimension were cut into individual strips and equilibrated in denaturing buffer (1% SDS and 1% *b*‐mercaptoethanol) for 1 h at RT and placed in a 2D SDS‐PAGE of the same thickness. Separation of the second dimension was performed in a 13% SDS‐polyacrylamide gel at 25 mA per gel. At the end of the run, the gel was blotted onto a PVDF membrane (Millipore) for Western blot analysis.

### 2.12 Western blotting and densitometric analysis

After transfer, the membranes containing the blotted proteins were blocked and incubated with primary antibodies diluted as described in Section 2.3. After washing, the membranes were incubated with peroxidase‐conjugated secondary antibodies and washed again. Reactive proteins were revealed with an enhanced chemiluminescent detection system (Clarity western ECL substrate, Bio‐Rad) and visualized on a Chemi‐Doc imaging system (Bio‐Rad). Images were recorded and data analyzed with Image lab software (Bio‐Rad). Actin or GAPDH were used as an internal control for protein loading. For the analysis of M1‐AQP4 expression in spinal cords, many animals, and membranes were analyzed. To pool together data obtained from different animals and filters, the analysis of each filter was carried out comparing the M1‐AQP4/actin ratio of heterozygous and M23‐null mice with the M1‐AQP4/actin ratio of the WT mice of the same filter. Data are expressed as a percentage of the M1‐AQP4/actin of the WT mice.

### 2.13 RNA extraction, RT‐PCR, and isoform‐specific qPCR

Tissues and cells RNA extraction was carried out using Trizol Reagent (Invitrogen) according to the instruction manual. Total RNA was quantified by Nanodrop (Thermo Scientific, Waltham, MA). Then, 3 μg of total RNA were retrotranscribed by SuperScript IV Reverse Transcriptase (Invitrogen). RT‐PCR was performed to assess the presence of AQP4‐Δ4 splice variant (de Bellis M, et al. 2014). AQP4 total expression and AQP4‐M1 mRNA isoform were analyzed by real‐time quantitative RT‐PCR using Power Syber Green and the StepOne Real‐Time PCR Detection System (Applied Biosystems, Milan, Italy). The isoform specific primers were AQP4‐tot for both isoform amplification and AQP4‐M1for specific M1‐AQP4 isoform amplification. The primers used are listed in Table 1. AQP4 total expression and AQP4‐M1 was measured by the GAPDH normalized ΔΔCt quantification method. All reactions were run in triplicate. After statistical analysis, the data from the different experiments were plotted and averaged in the same bar graph. Each PCR was evaluated by melting‐curve analysis.

**TABLE 1:** The isoform specific primer sequences

	FORWARD	REVERSE
AQP4‐M1	cagggaaggcatgagtgacag	cccacaccgagcaaaacaaag
AQP4‐tot	Cggttcatggaaacctcacc	catgctggctccagtataat
GAPDH	Tgcaccaccaatgcttagc	ggcatggactgtggtcatga

### 2.14 RNA‐protein pull‐down to isolate mouse M1‐AQP4 RBP from mouse spinal cord

Mouse spinal cord cDNA was obtained by First strand cDNA synthesis, using the SuperScript IV kit (ThermoFisher) with random esamers and amplified by M1‐AQP4 mRNA‐specific AQP4 primers to obtain a T7 promoter linked PCR.

Primers were designed to span 5′UTR, CDS and part of 3′UTR of mouse M1‐AQP4 mRNA. T7 mM1‐AQP4‐For: TAATACGACTCACTATAGGGGCCACATGGTGCAGAATCTTTC and mM1‐AQP4‐Rev: TTTAGACACCAACTAAAAGTC were used for this aim. T7 mGAPDH‐For: TAATACGACTCACTATAGGGATGGTGAAGGTCGGTGTGAA and mGAPDH‐Rev: TTACTCCTTGGAGGCCATGTAG were used to design PCR to synthetize capped full length GAPDH mRNA. The PCR products were purified using QIAEX II (Qiagen), fully sequenced and transcribed in a capped (^m7^G^5′^PPP^5′^G) mouse M1‐AQP4 mRNA or capped (^m7^G^5′^PPP^5′^G) mouse GAPDH mRNA using the mMESSAGE mMACHINE kit (Ambion). The capped mRNAs were purified using the RNeasy‐Mini Kit (Qiagen) and quantified with a NanoDrop spectrophotometer (ThermoFisher).

The capped M1‐AQP4 mRNA, the capped GAPDH mRNA and the negative control RNA from the Pierce RNA 3′ End Desthiobiotinylation Kit (PolyA) were biotinylated to the 3′ end and captured on streptavidin magnetic beads according to the manual instructions. Spinal cord samples were explanted and immediately extracted using 25 mM Tris–HCl pH 7.4, 150 mM NaCl, 1% NP‐40, 1 mM EDTA, 5% glycerol and Complete Roche protease inhibitor cocktail. Protein concentrations were obtained using the Protein‐BCA kit (Pierce). Spinal cord extracts (120 μg/each assay) were incubated with capped M1‐AQP4 mRNA‐coated, PolyA RNA or capped GAPDH mRNA‐coated and nude beads in the presence of di RNAse OUT (ThermoFisher) at a final concentration of 0.4 U/μl according to the Pierce Magnetic RNA‐Protein Pull‐Down Kit instructions. After the binding phase and washing steps, proteins were eluted from beads by biotin elution buffer provided in the same kit. Eluates were analyzed by SDS‐PAGE followed by silver staining (Mortz et al., 2001), by differential quantitative gel‐free mass spectrometry (MS) or by Western blotting.

In particular, eluates obtained from four mice for each genotype were first analyzed by SDS‐PAGE and silver staining to globally analyze the differences between the electrophoresis profile of eluates obtained using AQP4 mRNA‐coated beads versus nude beads and versus control RNA‐coated beads.

After the first analysis performed by SDS‐PAGE, two eluates for each genotype obtained by AQP4 mRNA‐coated beads and control RNA‐coated beads were pooled and analyzed by differentially quantitative MS analysis (see next paragraph).

After the MS analysis, eluates obtained from another independent RNA‐protein pull down experiment, in which other two mice for each genotype were used, were analyzed by Western blotting.

To further validate the specificity of the binding to AQP4 mRNA, another independent RNA‐protein pull down experiment, followed by Western blotting analysis, were performed using six other mice and using the capped GAPDH mRNA as a negative control.

The number of animals is indicated in the figure legends for each type of analysis. All animals were age‐matched.

### 2.15 Quantitative MS of RNA‐protein pull down eluates

The MS analysis was performed by Ceinge Biotecnologie Avanzate (https://www.ceinge.unina.it/home).

Eluates from M1‐AQP4 mRNA‐ and PolyA RNA‐coated beads obtained by two WT and two M23‐null mice spinal cord were pooled and analyzed by S‐Trap digestion, LC MS/MS and MaxQuant analysis. Two LC MS/MS runs for each sample were performed.

Briefly, eluates were first mixed with SDS and DTT, boiled, cooled to RT, and then alkylated with iodoacetamide in the dark for 30 min. Subsequently, phosphoric acid was added to the samples with a final concentration of 1.2% and then the sample was diluted with six volumes of binding buffer (ammonium bicarbonate and methanol). After gentle mixing, the protein solution was loaded onto an S‐Trap filter, spun at 2000 rpm, and the flow‐through collected and reloaded onto the filter. This step was repeated three times, and then the filter was washed three times with binding buffer. Finally, the digestion buffer containing trypsin at 1:10 wt:wt was added onto the filter and digestion was carried out for 1 h. The peptide solution was pooled, lyophilized, and resuspended in 0.2% formic acid.

The tryptic peptides were analyzed by LTQ XL Orbitrap XL with ETD nano LC MS/MS LIT‐FITR (Thermo Fisher Scientific). After loading, the peptide mixture was first concentrated and desalted on the precolumn (C18 Easy Column L02 cm, ID = 100MM). Each peptide sample was fractioned on a C18 reverse phase capillary column (C18 Easy Column L020cm, ID = 7.5 Μm, 3 μm) working at a flow rate of 250 nl/min. The gradient used for peptide elution ranged from 5 to 95% of buffer B (ACN LC–MS Grade and HCOOH) in 300 min.

The raw files obtained from this analysis were used as input in the MaxQuant software. For protein identification, the raw data obtained from the LC–MS/MS analysis were entered into the MaxQuant software, using the MS/MS Ion Search option to search the Andromeda protein database. Andromeda is integrated into MaxQuant as well as the viewer application for inspection of raw data and identification of results. The LC–MS/MS raw data are used by MaxQuant for the identification step using the Andromeda database by matching peaks in the spectra to the theoretical fragment masses. The MS proteomics data have been deposited to the ProteomeXchange Consortium via the PRIDE (Perez‐Riverol et al., 2019) partner repository with the dataset identifier PXD024792.

### 2.16 In silico mRNA analysis for alternative open reading frames, IRES, RBP sites, and G‐RNA quadruplexes

The NCBI reference sequence of mouse M1‐AQP4 mRNA (NM_009700.3) was analyzed using free on‐line tools: open reading frame (ORF) finder, mFOLD, IRESPred, RBPsite, and QGRS. Alternative ORFs were identified by ORF finder, mFold was used to predict secondary mRNA structures. IRESPred was used to predict internal ribosome entry sites (IRES), RBPsite to identify RBPs binding sites and finally QGRS to predict G‐RNA quadruplexes structures. The websites used are reported as follows: https://www.ncbi.nlm.nih.gov/orffinder/; http://unafold.rna.albany.edu/?q=mfold; http://196.1.114.46:1800/IRESPred/home.html; http://rbpmap.technion.ac.il/.

### 2.17 Fluorescence‐quenching assay

Cell‐volume changes in control siRNA and DDX17 siRNA‐treated astrocyte primary cultures were measured by calcein‐quenching fluorescence assay, as described previously (Mola et al., 2009; Mola et al., 2016).

Cells were seeded on black, clear bottom 96‐well‐plates (Corning, New York, NY) coated with PDL at a density of 15,000 cells per well and analyzed 5 days after transfection. Cells were loaded with 10 μM membrane permeable calcein‐AM (Molecular Probes, Eugene, OR) at 37°C for 45 min in growth medium and then rinsed in isotonic PBS. Cytosolic calcein fluorophore exhibits concentration‐dependent quenching by intracellular components (proteins or salts) so that measured changes in fluorescence were directly proportional to changes in cell volume.

Calcein fluorescence signal was recorded on a Flex Station3 plate reader equipped with a liquid handling module (Molecular Devices, San Jose, CA) able to analyze real‐time fluorescence kinetic data in the 96‐well format (de Bellis et al., 2020). Fluorescence was excited at 490 nm and detected at 520 nm using dual monochromators.

The hypotonic challenge was applied 20 s after the beginning of data acquisition by adding an appropriate volume of NaCl‐free DPBS to achieve a 60 mOsm/L osmotic gradient. The fluorescence signal increases following the hypotonic stimulus due to cell swelling and then decreases during the regulatory volume decrease (RVD) which tends to restore the isosmotic condition. Each well was read continuously over a 100 s period to record both the swelling phase and RVD phase.

Data acquisition was performed using SoftMax Pro software, and the data were analyzed with Prism software (GraphPad Software, La Jolla, CA). Graphs were obtained by fitting the data with an exponential function. The percentage of volume recovery was calculated from the maximum intensity of fluorescence reached after the osmotic shock (the amplitude of cell volume variation) and the level of fluorescence reached after the regulatory mechanism.

### 2.18 Statistical analysis

Statistical analyzes were conducted using GraphPad Prism 6 software. All data are reported as the mean ± *SEM*. We used the Student's *t* test for unpaired data and the one‐way ANOVA with Tukey's multiple comparisons test to compare more than two groups. Differences were considered significant for *p* < .05.

## 3 RESULTS

### 3.1 M23‐AQP4 deletion affects OAPs assembly, perivascular localization, and M1‐AQP4 expression in mouse spinal cord

**FIGURE 1:**
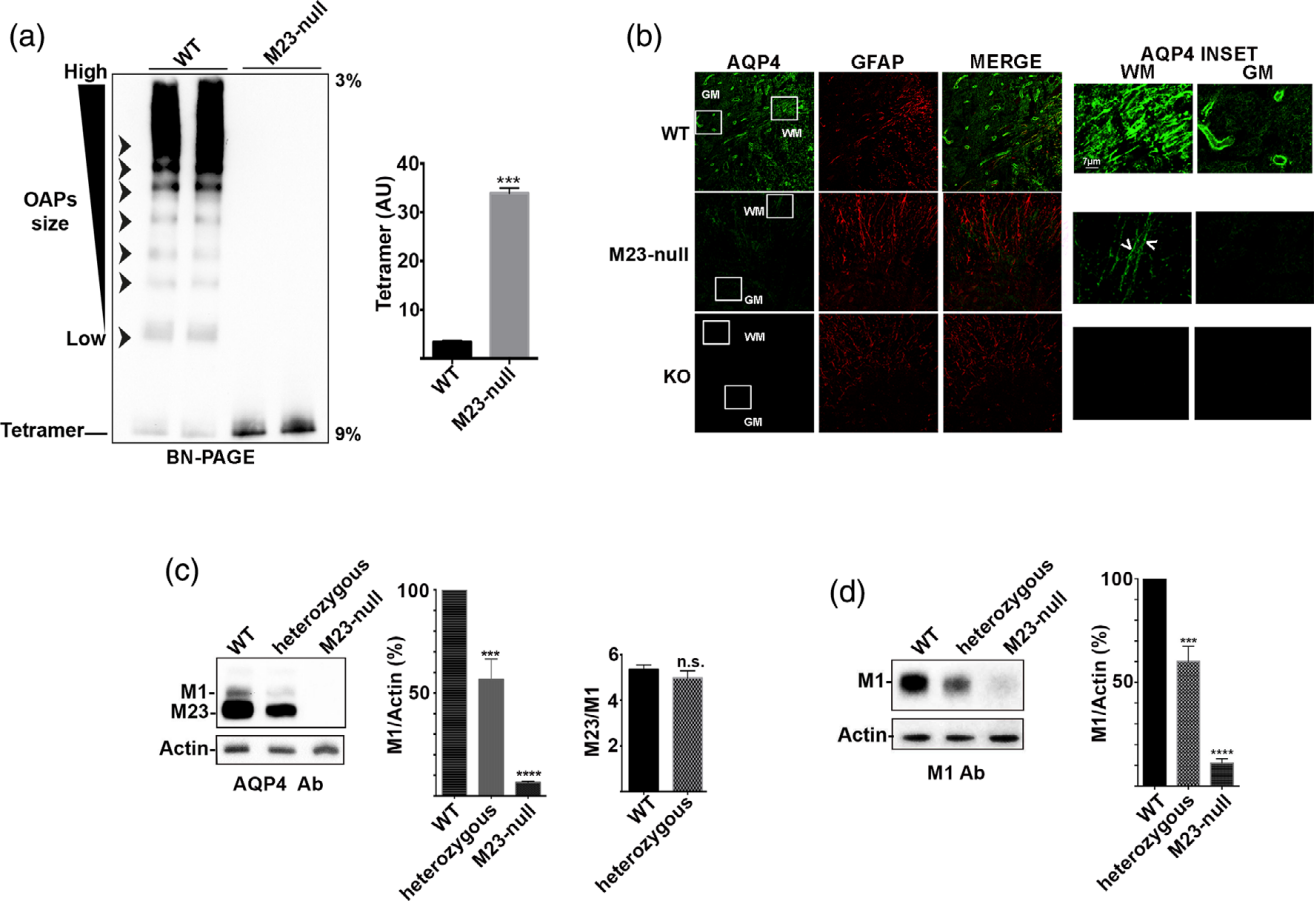
Orthogonal arrays of particles (OAP) assembly, aquaporin‐4 (AQP4) localization, and M1/M23 expression in WT, heterozygous and M23‐null spinal cord. (a) Right, BN/PAGE analysis of AQP4 supramolecular assembly in WT and M23‐null mice spinal cord. OAPs of different sizes are visible in WT, while they are completely absent in M23‐null mice, indicated by arrow heads. Left, densitometric analysis of AQP4 tetramer. Tetramer is strongly upregulated in M23‐null mice. ***p < .001 M23‐null versus WT. *n* = 4 for each genotype. Student's *t* test for unpaired data. (b) AQP4 (green) and GFAP (red) localization in mouse spinal cord sections analyzed by single scan confocal microscopy. In WT, AQP4 is strongly expressed at both protoplasmic astrocytes of gray matter (GM) and fibrous astrocytes of white matter (WM). In M23‐null mice, the AQP4 signal is strongly reduced in both sites and the perivascular staining is completely absent. M23‐null mice only show low AQP4 staining in fibrous astrocytes of white matter (arrowheads). *n* = 6 for each genotype. No staining was observed in AQP4 KO mice spinal cord sections. (c) Western blotting analysis of M1‐AQP4 and M23‐AQP4 expression using C‐terminal specific antibody. Densitometric analysis of M1‐AQP4 expression (middle), and M23/M1 ratio (right). Data were analyzed by one‐way ANOVA with Tukey's multiple comparisons test for M1/actin (%) and Student's *t* test for unpaired data for M23/M1 ratio analysis. ***p < .001 versus WT; ****p < .0001 versus WT; n.s. not statistically significant. Number of animals: WT (*n* = 6), heterozygous (*n* = 3), M23‐null (*n* = 6). (d) Western blotting analysis of M1‐AQP4 expression using M1‐AQP4‐specific antibody. Densitometric analysis of M1‐AQP4 expression using M1‐AQP4‐specific antibody (right). Data are reported as a percentage of M1‐AQP4 expression considering the M1‐AQP4 expression in WT as 100% and analyzed by one‐way ANOVA with Tukey's multiple comparisons test. ***p < .001 versus WT; ****p < .0001 versus WT; n.s. not statistically significant. Number of animals: WT (*n* = 6), heterozygous (*n* = 3), M23‐null (*n* = 6). Samples from the same animals were analyzed both with C‐terminal specific and with M1‐AQP4‐specific antibodies [Color figure can be viewed at wileyonlinelibrary.com]

AQP4 supramolecular assembly state in WT and M23‐nullspinal cord was initially analyzed by BN/PAGE (Figure 1(a), left). In WT, OAPs of different size can be detected (arrowheads in Figure 1(a), left), while in M23‐null, only the tetrameric form of AQP4 is visible and OAPs are completely absent. In spinal cord of M23‐null mice, the tetrameric form is upregulated compared to WT (Figure 1(a), right), in agreement with the results we have recently reported in brain (de Bellis et al., 2020).

To investigate whether the M23‐AQP4 absence affects AQP4 localization in spinal cord, AQP4 and GFAP co‐immunofluorescence was performed followed by confocal microscopy analysis using anti‐C‐terminal AQP4 antibody as primary antibody. The single scan confocal microscopy shows a strong AQP4 signal in gray and white matter of WT (Figure 1(b)). In M23‐null gray matter, the perivascular AQP4 was not detected. Interestingly, in M23‐null, the residual AQP4 appears to be confined to the fibrous astrocytes of the white matter (Figure 1(b), arrowheads in the magnified inset). No staining was detected in spinal cord sections of AQP4 KO mice.

To analyze the possible correlation between expression levels of M23‐AQP4 and those of M1‐AQP4, we performed SDS‐PAGE and Western blot analysis in WT, heterozygous, and M23‐null mice. We used two different antibodies, one directed against the AQP4 C‐terminal, therefore able to recognize both isoforms (Figure 1(c)), and the other one specific for the M1‐AQP4 isoform (Figure 1(d)). Densitometric analysis of blots obtained with these two antibodies shows a 40–50% downregulation of M1‐AQP4 expression in heterozygous and a 90–95% downregulation in M23‐null compared to WT mice (Figure 1(c) middle and Figure 1(d), right). Interestingly, using the anti C‐terminal antibody we show that in heterozygous mice the M23/M1 ratio remains unchanged compared to WT (Figure 1(c), right), despite the M1‐AQP4 isoform being significantly reduced (Figure 1(d)). This suggests that M1‐AQP4 and M23‐AQP4 isoforms are probably regulated in an interdependent way.

### 3.2 M1‐AQP4 mRNA levels, AQP4 mRNA alternative splicing, and M1‐AQP4 protein degradation are unaffected in M23‐null spinal cord and the absence of M23‐AQP4 does not destabilize M1‐AQP4 protein in astrocyte primary culture

**FIGURE 2:**
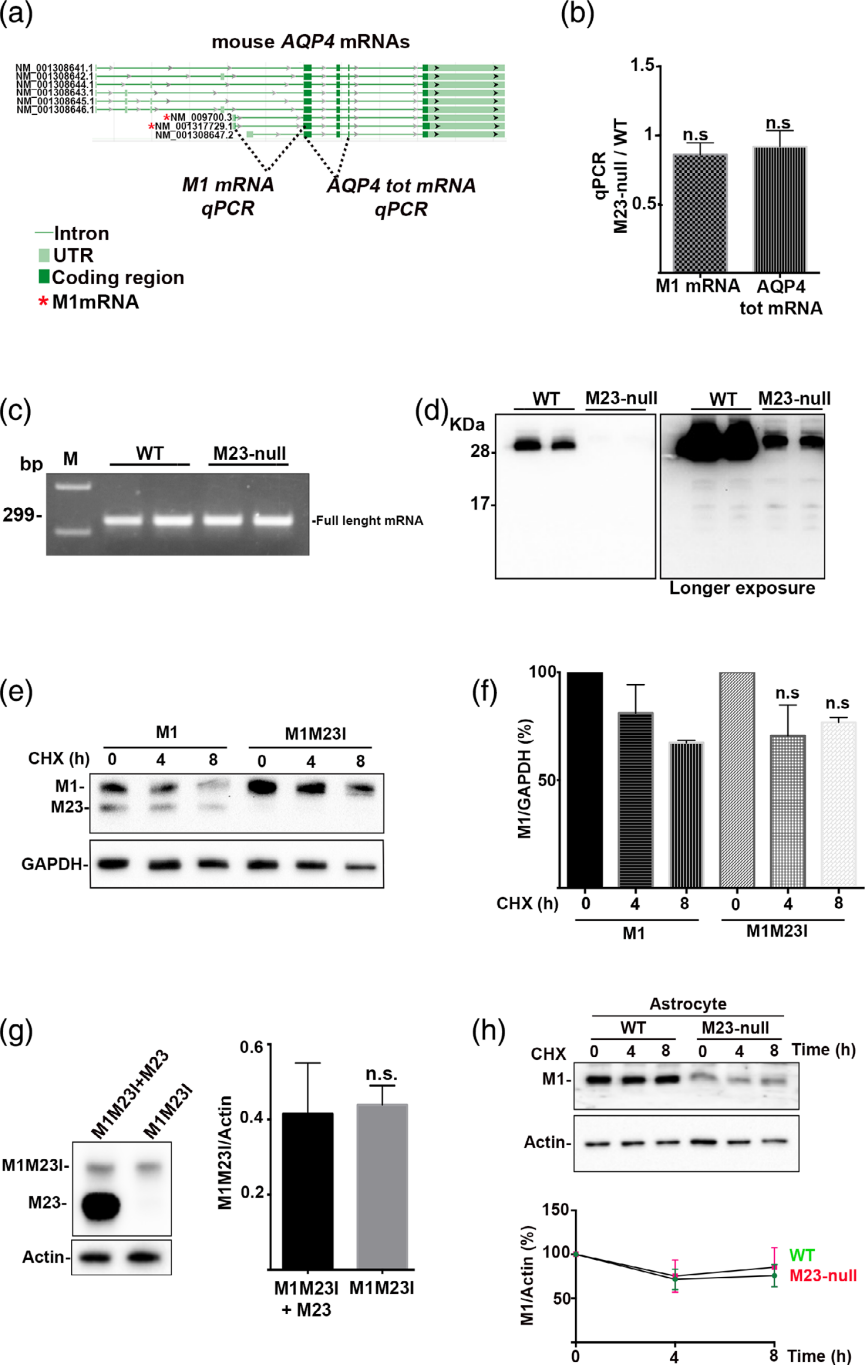
Analysis of M1‐aquaporin‐4 (AQP4) mRNA levels, alternative splicing and posttranslational control of *Aqp4* gene in M23‐null mice spinal cord. (a) Reference sequence details of mouse AQP4 transcripts and schematic position of total‐ and M1‐AQP4‐mRNA‐specific primers used in qPCR. Sequences obtained by NCBI (Gene ID: 11829). (b) AQP4‐Isoform specific qPCR analysis in spinal cord of WT and M23‐null mice. No differences in M1‐AQP4 mRNA or AQP4‐tot mRNAs levels were observed between WT and M23‐null. *n* = 4 for each genotype. n.s. not statistically significant M23‐null*vs* WT. Student's *t* test for unpaired data. (c) AQP4‐Δ4 specific RT‐PCR in WT and M23‐null mice. No trace of Δ4 isoform (218 bp) was observed in spinal cord, only the full‐length mRNA (299 bp) was detected. *n* = 4 for each genotype. (c) Analysis of AQP4‐protein degradation in spinal cord of WT and M23‐null mice. Early‐stopped SDS‐PAGE and Western blotting showed no sign of AQP4 degradation in M23‐null mice. *n* = 4 for each genotype. (e) Analysis of M1M23I protein stability in transfected HEK cells. Cycloheximide (CHX)‐treated M1 and M1M23I expressing cells were analyzed for M1‐AQP4 protein expression at 0, 4, and 8 h after CHX treatment as indicated in each lane. (f) Densitometric analysis of Western blotting reported in Panel (e). No difference in M1‐AQP4 protein posttranslational stability was observed between WT (M1‐AQP4) and mutated (M1M23I) protein. *n* = 3 for each time point, n.s. not statistically significant M1M23I versus M1 for each time point. Student's *t* test for unpaired data. (g) Analysis of M1M23I protein expression in the presence of M23‐AQP4 isoform in co‐transfected HEK cells. HEK cells were co‐transfected with five parts of M23‐AQP4 and with one part of M1M23I, to mimic the isoform ratio observed in WT spinal cord. The empty vector was used as control DNA to equalize the DNA amount. No sign of M1M23I protein stabilization was observed in the presence of M23‐AQP4 isoform. *n* = 4–6 for each condition. Student's *t* test for unpaired data. (h) Cycloheximide (CHX) assay analysis of M1‐AQP4 posttranslational stability in WT and M23‐null astrocyte primary culture. WT and M23‐null astrocyte were treated with 30 μM CHX for 0, 4, and 8 h and M1‐AQP4 was measured by Western blotting. No difference in the M1‐AQP4 stability was observed between WT and M23‐null astrocyte. *n* = 3 for each condition. Student's *t* test for unpaired data [Color figure can be viewed at wileyonlinelibrary.com]

We have already shown that total AQP4 mRNA level is unchanged between WT and M23‐null brain (de Bellis et al., 2020). Those data were obtained using qRT‐PCR primers specific for the exon‐junction II‐III and for Exon IV. Considering that exons II‐III and IV are identical in many different mRNAs able to express M23‐ and M1‐proteins (NCBI Gene ID: 11829), the data obtained using the primers described above were not a direct measure of M1‐mRNA. Here, to test the hypothesis of transcriptional control as a cause of M1 protein downregulation, WT and M23‐null mice spinal cords were analyzed by M1‐AQP4‐specific qPCR. To this purpose an M1‐ and an AQP4‐tot‐specific qPCR were designed (Table 1) based on the reference sequence reported in the NCBI RefSeq Database (Figure 2(a)). As indicated in Figure 2(b), no difference was found between WT and M23‐null for either M1‐AQP4 or total mRNAs. Considering that the amplification efficiency of both pairs of primers is identical (Supplementary Figure 1) and that the Ct of AQP4 tot is always one cycle lower compared to the Ct of M1‐AQP4 mRNA in both WT and M23‐null (Supplementary Figure 2), we conclude that the M1mRNA is not the only AQP4 mRNA expressed in the spinal cord.

To test the hypothesis of alternative splicing as a cause of M1‐downregulation, Δ4‐specific RT‐PCR was performed as previously described (De Bellis et al., 2014). Only the 299 bp RT‐PCR product, specific for Exon IV‐containing mRNAs, was detected while no evidence of Δ4‐specific RT‐PCR product of 218 bp was observed (Figure 2(c)). This excludes the presence of AQP4‐Δ4 mRNA in both WT and M23‐null spinal cord. To assess whether the absence of M23‐AQP4 induces the M1‐AQP4 degradation, independently of the presence of AQP4‐Δ4, a Western blot analysis of WT and M23‐null spinal cord after a short SDS‐PAGE run was performed to detect potential smaller products of M1‐AQP4 degradation in M23‐null mice. No protein products of M1‐AQP4 degradation were observed in M23‐null samples (Figure 2(d)). Considering that the M23‐null mouse model expresses the mutated form of the M1‐AQP4 isoform (M1M23I) and considering that some mutations in AQP4 CDS could affect AQP4 expression (Pisani, Mastrototaro, et al., 2011), we tested the hypothesis that the mutation (M23 > I) could be able to affect M1‐AQP4 protein stability. To measure the posttranslational stability of M1M23I versus M1 WT, CHX chase assay was performed in HEK cells transfected with WT and M1M23I isoforms. As reported in Figure 2(e,f), the stability of M1M23I is comparable to that of M1 WT protein in transfected HEK cells. To assess whether M1M23I expression was in some way stabilized by the co‐presence of M23‐AQP4, we analyzed M1M23I abundance in M1M23I and M1M23I + M23‐AQP4 co‐transfection experiments in HEK cells. To mimic the isoform ratio observed in WT spinal cord (Figure 1(c)), we used one part of M1M23I coding plasmid and five parts of M23 expressing plasmid (or an empty vector control plasmid). The results obtained indicate that M1M23I protein expression was unchanged by the presence of M23‐AQP4 in transfected cells (Figure 2(g)). To test the effect of the M23‐AQP4 absence in the M1‐AQP4 posttranslational stability in naturally AQP4 expressing cells, CHX chase assay was performed using WT and M23‐null astrocyte primary culture. The data also show that the absence of M23‐AQP4 does not affect M1‐AQP4 posttranslational stability in primary cell culture that naturally express AQP4 (Figure 2(h)).

### 3.3 RNA‐protein pull down and differential quantitative MS analysis of M1 RBPs in spinal cord of WT and M23‐null mice

**FIGURE 3:**
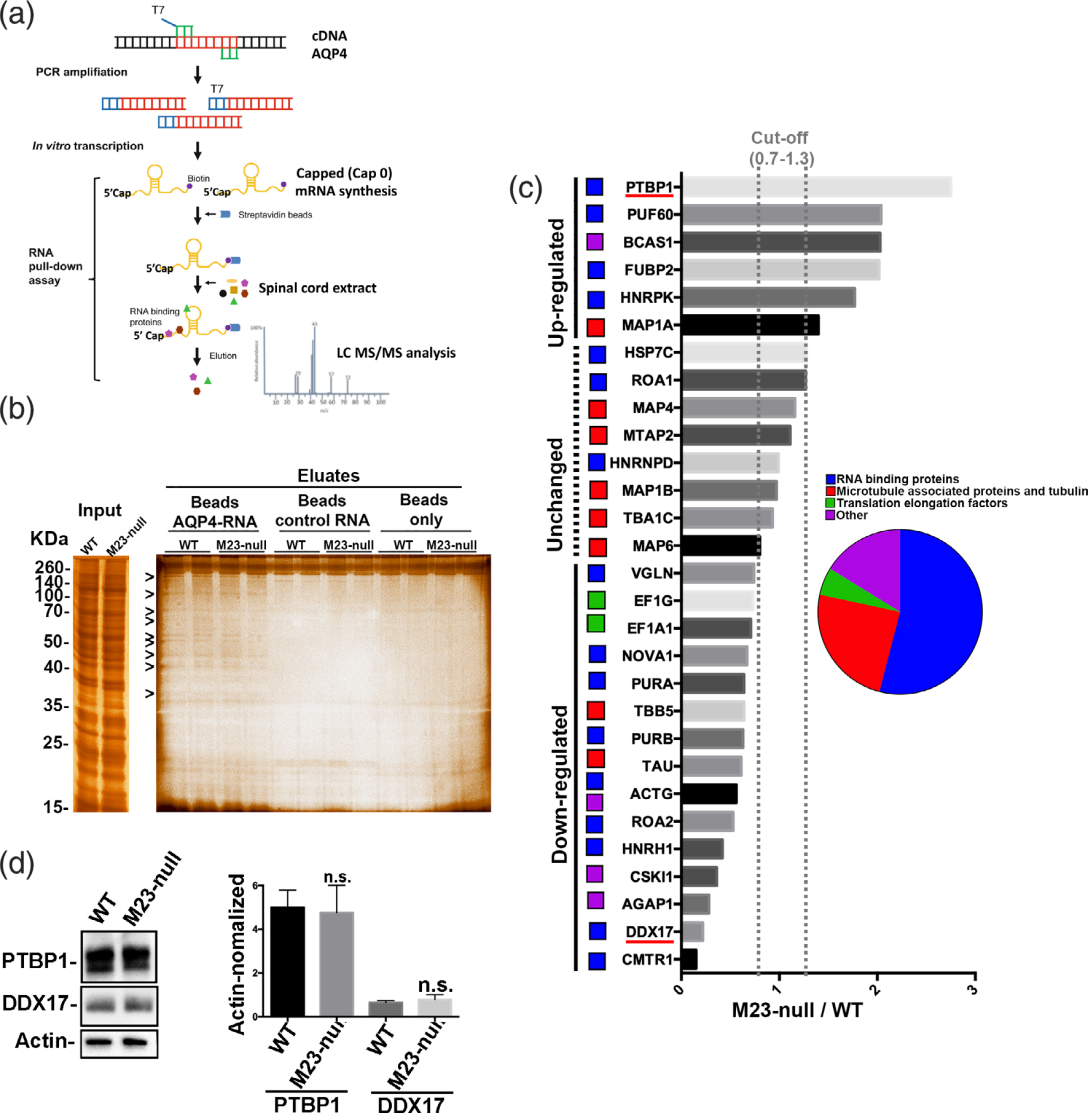
Aquaporin‐4 (AQP4)‐translational control in mouse spinal cord explored by RNA‐protein pull down and quantitative LC mass spectrometry (MS)/MS analysis. (a) Overview of RNA‐protein assay. The spinal cord cDNA was amplified by M1‐AQP4 mRNA‐specific AQP4 primers to obtain a T7 promoter linked PCR. Primers were designed to span 5′UTR, CDS and part of 3′UTR of M1‐AQP4 mRNA. The PCR product was transcribed in a capped (^m7^G^5′^PPP^5′^G, similar to the Cap0 structure) mRNA. The capped M1‐AQP4 mRNA was biotinylated to the 3′ end and captured on streptavidin magnetic beads. Spinal cord extracts from M23‐null and WT mice were incubated and eluates identified by LC MS/MS analysis. Negative control RNA coated beads and nude beads were used as control conditions. Eluates from control RNA coated beads were also identified and quantified by MS to identify non‐specific binders, eliminated from the subsequently analysis. (b) Representative SDS‐PAGE and silver staining analysis of spinal cord input and eluates from AQP4‐RNA coated, control‐RNA coated and nude beds. Arrowheads indicate bands specific for AQP4‐RNA coated beads. *n* = 4 for each genotype. (c) Quantitative differential MS analysis of AQP4‐mRNA eluates in M23‐null versus WT. Only proteins specific for AQP4‐RNA coated beads are shown in the graph. These proteins were identified as M1‐AQP4‐mRNA binders (M1‐AQP4‐RBPs). The quantitative differential analysis of M1‐AQP4‐RBPs abundance in M23‐null and WT mice is shown as the M23‐null/WT ratio. The unchanged proteins are in the range of 1 ± 0.3. The group of M1‐AQP4‐RBPs found to be more abundant in the M23‐null eluates is indicated as upregulated while the group of M1‐AQP4‐RBPs found to be less abundant in the M23‐null eluates is indicated as downregulated. PTBP1 was found to be 2.75‐fold more abundant in KI (M23‐null/WT = 2.75, red highlighted) while DDX17 and CMTR1 were found to be five to six times less abundant in M23‐null (M23‐null/WT = 0.22 for DDX17 and 0.15 for CMTR1, highlighted in red). *n* = 2 for each genotype, data were obtained using pooled samples. (d) Western blotting analysis of PTBP1 and DDX17 in WT and M23‐null mice spinal cord showed no changes in expression levels of both proteins. *n* = 7 for each genotype [Color figure can be viewed at wileyonlinelibrary.com]

Starting from the evidence that AQP4 transcription, splicing and protein degradation were unaffected in M23‐null mice and in M23‐null astrocyte primary culture, we investigated the translational control. Considering that RBPs actively control mRNA translation in vivo, we identified and quantified RBPs able to bind M1‐AQP4 mRNA in WT and M23‐null mice spinal samples. RNA‐protein pull down assay followed by quantitative differential (M23‐null vs. WT) LC MS/MS MS analysis (differential quantitative MS [dQMS]) were used for this scope (Figure 3(a)).

Eluates from M1‐AQP4 mRNA‐coated, control RNA‐coated, and nude‐beads were first analyzed by SDS‐PAGE followed by silver staining. Specific bands were identified for M1‐AQP4 mRNA‐coated beads (Figure 3(b), arrowheads). Eluates from M1‐AQP4 mRNA‐ and negative control RNA‐coated beads obtained by WT and M23‐null spinal cord were then analyzed by dQMS. AQP4 RBPs (AQP4‐RBPs) were identified as proteins able to bind AQP4 mRNA coated bead and unable to bind the control coated beads (Supporting information and raw data deposited in PRIDE database [dataset identifier PXD024792]). Data obtained by dQMS are expressed as the M23‐null/WT ratio for each AQP4 mRNA RBP (Figure 3(c)). Most M1‐AQP4 mRNA RBP have already been described as canonical RBPs (Figure 3(c), blue boxes).

We found that M1‐AQP4 mRNA interacts with microtubule and tubulin‐associated proteins (Figure 3(c) red boxes) which however remain mainly unchanged between WT and M23‐null mice. Interestingly, compared to WT, PTBP1, previously demonstrated as a positive regulator of M23‐AQP4 translation (Baird et al., 2007), was the most abundant M1‐AQP4 mRNA interactor in eluates from M23‐null (M23‐null/WT ratio 2.75, highlighted in red in Figure 3(c)), while CMTR1 and DEAD‐box RNA helicases 17 (DDX17) were the least abundant in eluates from M23‐null (M23‐null/WT ratio 0.22 for DDX17 and 0.15 for CMTR1, highlighted in red in Figure 3(c)). Western blotting analysis performed by anti‐PTBP1 antibody of RNA‐protein pull‐down eluates obtained using other WT and M23‐null mice support data obtained by dQMS (Supplementary Figure 3(a)).

To further test the binding specificity for M1‐AQP4 mRNA, RNA‐protein pulldown was performed using other mice spinal cord samples and full length capped GAPDH mRNA as negative control. Western blotting analysis of eluates confirms the binding specificity for M1‐AQP4 mRNA (Supplementary Figure 3(b)).

To analyze the expression levels of PTBP1 and DDX17 in WT and M23‐null mice spinal cord, RNA‐protein pull‐down inputs were analyzed by Western blotting. The data show that expression levels of both proteins were unchanged in M23‐null mice respect those measured in WT mice (Figure 3(d)).

### 3.4 Functional validation of M1‐AQP4 mRNA RBPs in the regulation of AQP4 expression and cell swelling by RNAi in astrocyte primary cultures

**FIGURE 4:**
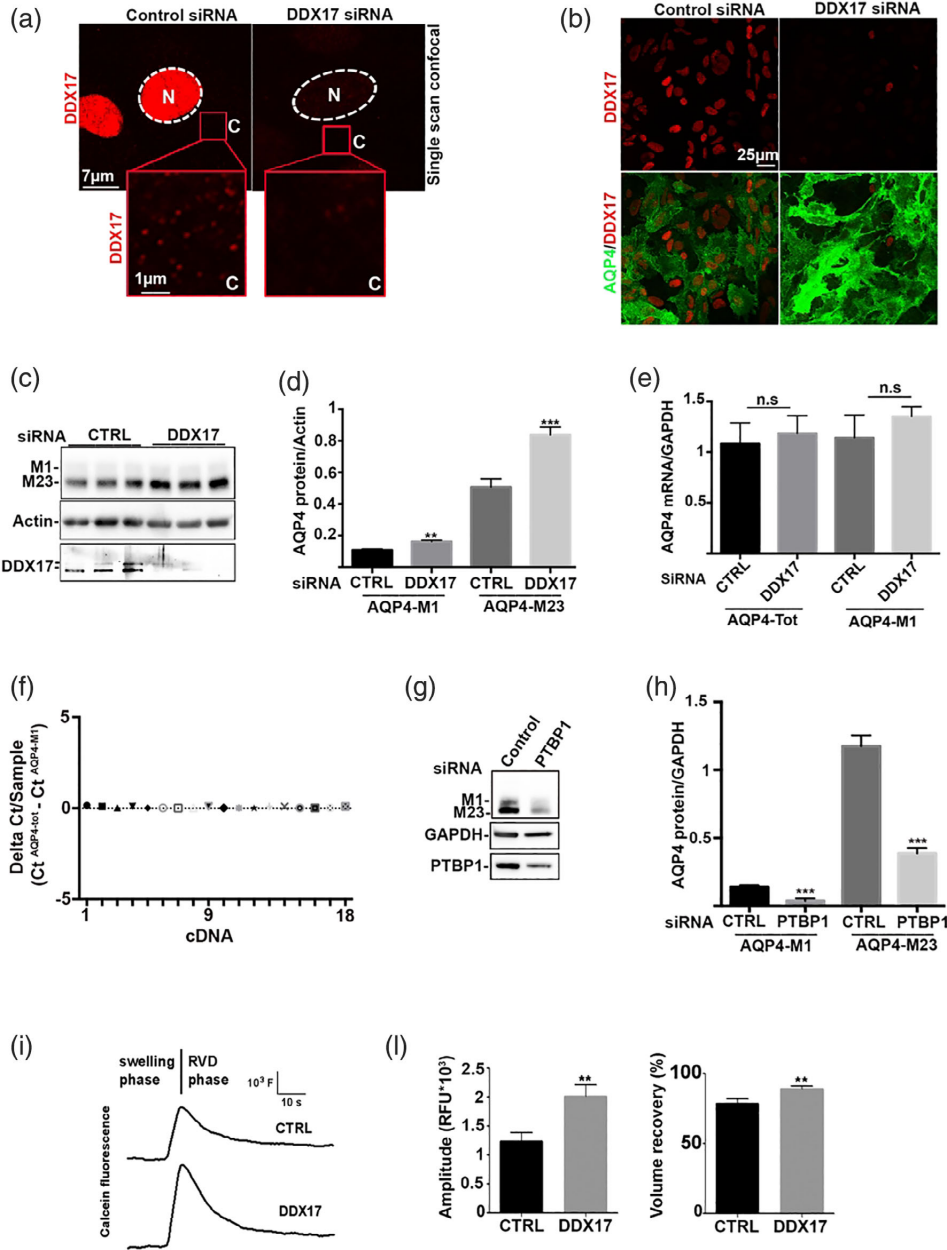
Functional validation of DDX17 and PTBP1 in the aquaporin‐4 (AQP4) regulation by RNAi in mouse astrocyte primary cultures. (a) Confocal microscopy analysis of DDX17 (red) expression in control siRNA and DDX17 siRNA‐treated astrocyte primary cultures. The immunofluorescence shows the main localization of DDX17 into the nucleus (N) and a lower dot‐like cytoplasmic staining (C), enlarged in the red boxed area. (b) Wide‐field immunofluorescence analysis of AQP4 (green) and DDX17 (red) shows a strong reduction in DDX17 staining in DDX17 RNAi treated astrocytes and the upregulation of AQP4 compared with control siRNA‐treated astrocytes. (c) Representative Western blotting analysis of astrocytes treated with control and DDX17 siRNA. DDX17 is strongly downregulated while AQP4 expression is upregulated in DDX17 siRNA conditions. *n* = 9 from three independent astrocytes preparations. (d) Densitometric analysis of Western blotting represented in Panel (c). DDX17 knockdown strongly upregulates AQP4 expression. **p = .005, ***p = .0001, DDX17 siRNA versus CTRL siRNA; *n* = 9 from three independent astrocytes preparations. Student's *t* test for unpaired data. (e) Relative quantification of AQP4‐tot and AQP4‐M1 mRNA between control‐ and DDX17‐siRNA‐treated astrocytes obtained by RT‐qPCR from the same samples reported in Panels (c) and (d). No differences were found for either target. *n* = 9 from three independent astrocyte preparations. Student's *t* test for unpaired data. (f) ΔCt (Ct AQP4‐tot) ‐ (Ct AQP4‐M1‐AQP4) analysis using cDNA from mouse astrocyte samples (*n* = 18 from three independent astrocyte preparations). (g,h) Functional validation of PTBP1 in the AQP4 regulation by RNAi in mouse astrocyte primary cultures. Astrocyte primary cultures treated with control and PTBP1 siRNA analyzed by Western blotting (g) and densitometric analysis (h). PTB knockdown strongly reduces AQP4 expression, ***p < .0001, PTBP1 siRNA versus CTRL siRNA; *n* = 10 from three independent astrocyte preparations. Student's *t* test for unpaired data. (i**–**l) Calcein‐quenching assay for measurement of hypotonicity‐induced volume changes in cultured WT astrocytes treated with scramble siRNA (CTRL) and DDX17 siRNA. (i) Representative time course of cell swelling (swelling phase) followed by regulatory volume decrease (regulatory volume decrease [RVD] phase) recorded from calcein‐loaded cells upon exposure to 60 mOsm/L hypotonic gradient. (l) Histogram showing the means ±ES values of the magnitude of cell swelling and of the extent of volume recovery (in percent) obtained from 14 to 18 measurements of three set of independent experiments. Note that DDX17 knockdown enhanced cell swelling amplitude and the efficiency of RVD. Statistical analysis was performed by *t* test for unpaired data (**p < .001) [Color figure can be viewed at wileyonlinelibrary.com]

The RNA‐protein pull down and the quantitative MS indicate the AQP4 RBPs DDX17, CMTR1, and PTBP1 as possible candidates involved in the AQP4 translational control in response to the M23‐AQP4 deletion in mouse CNS. To explore the role of DDX17 in the regulation of AQP4 expression, we used astrocyte primary cultures that naturally express AQP4. Immunofluorescence analysis shows that astrocytes express high levels of DDX17 in the nucleus and laser‐scan confocal microscopy shows that DDX17 is also expressed in the cytoplasm as dot‐like staining (red squares in Figure 4(a)). DDX17 knockdown reduced both nuclear and cytoplasmic staining (Figure 4(a)). Immunofluorescence (Figure 4(b)) shows the strong reduction of DDX17 and the upregulation of AQP4 in DDX17 siRNA‐treated astrocytes. Western blotting and densitometric analysis (Figure 4(c,d)) show that DDX17 knockdown strongly upregulates AQP4 expression, while GFAP, PTBP1, and GAPDH were unaffected by DDX17 knockdown (Supplementary Figure 4). Interestingly, AQP4‐qPCR analysis of cDNA prepared from the same samples shows that DDX17 knockdown does not change either AQP4‐tot mRNAs or M1‐AQP4 mRNA levels (Figure 4(e)). Furthermore, the amplification efficiency of AQP4‐tot‐ and AQP4‐M1‐AQP4 mRNA‐specific primers are identical (Supplementary Figure 1) and no differences emerge from AQP4‐tot Ct and AQP4‐M1‐AQP4 mRNA Ct in astrocytes (Figure 4(f)). This indicates that astrocyte primary culture only expresses M1‐AQP4 mRNA.

The role of PTBP1 as a positive M23‐AQP4 translational regulator had previously been described in the literature by in silico prediction and luciferase assay in transfected cells (Baird et al., 2007). We found that the knockdown of PTBP1 in astrocyte primary cultures reduced both M23‐AQP4 and M1‐AQP4 expression (Figure 4(f,g)). Due to a basal very high level of AQP4 expression and water transport rate in WT astrocytes (Mola et al., 2016), swelling analysis in response to hypotonic shock of DDX17 knockdown astrocytes resulted in an increased magnitude of cell swelling and cell volume recovery during the RVD phase (Figure 4(i–l)). Finally, CMTR1, the first transcribed nucleotide ribose 2′‐*O* methyltransferase (Inesta‐Vaquera & Cowling, 2017), which is the RBP that exhibits the lowest binding to AQP4‐M1 mRNA in M23‐null compared to WT (Figure 4(c)), was not here tested by RNAi because it is a key enzyme involved in the 5′CAP‐mRNA synthesis and, as already shown (Inesta‐Vaquera & Cowling, 2017), its knockdown could affect the 5′CAP‐dependent protein translation of all genes.

### 3.5 In silico analysis for M1‐AQP4 mRNA regulatory elements

**FIGURE 5:**
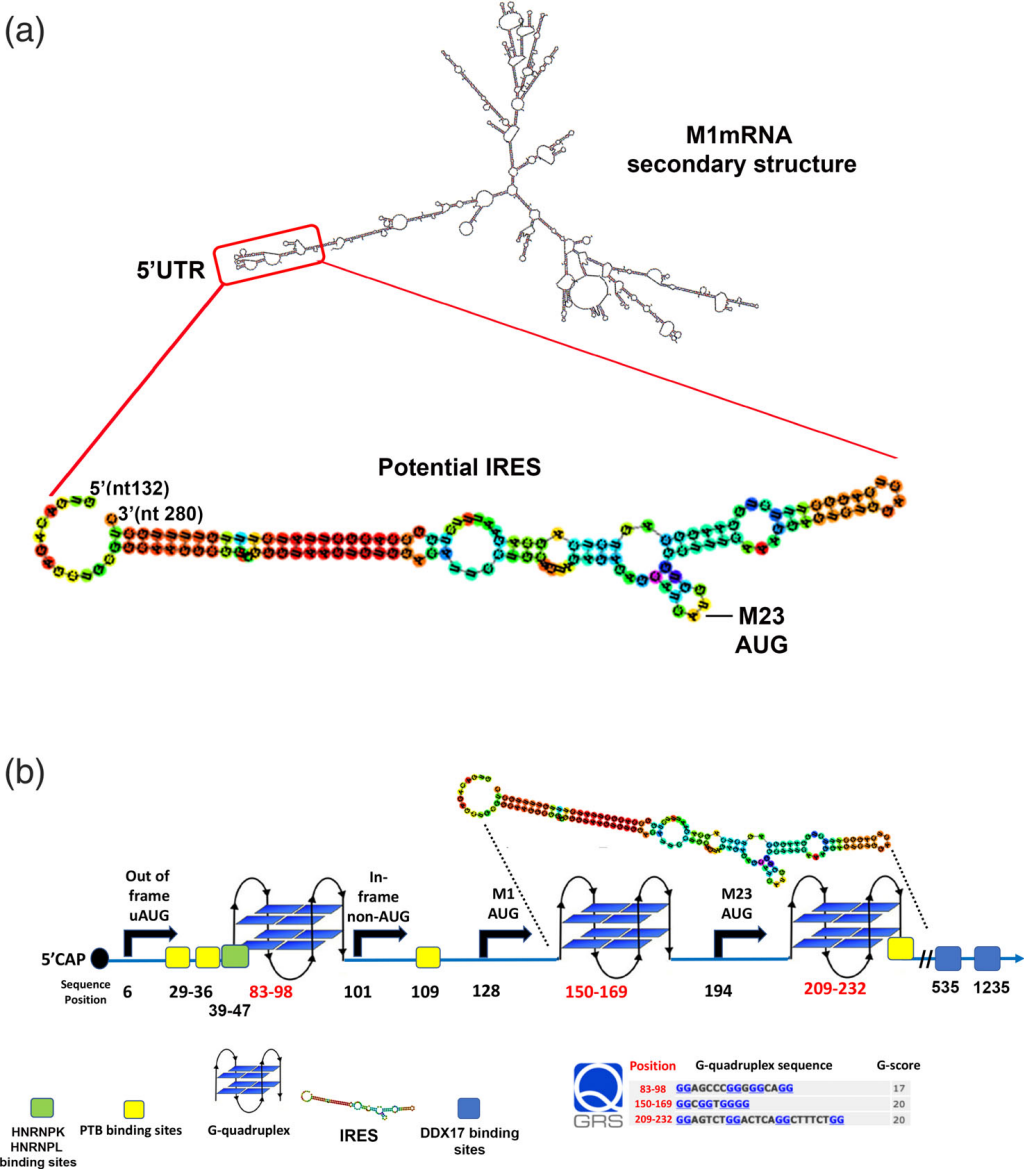
Searching for M1‐aquaporin‐4 (AQP4) mRNA regulatory elements and protein binding sites. (a) mFOLD prediction of mouse M1‐AQP4 mRNA secondary structure and IRESPred IRES prediction. The most stable structure is reported. The red box highlights the 5′UTR which is potentially able to form a very stable stem‐loop structure containing both M1‐AQP4 and M23‐AQP4 translation initiation signals (TISs). Notably, IRESPred indicates the presence of a potential IRES between nucleotides 132 and 280. (b) Detailed analysis performed using open reading frame (ORF) finder, RBPsite, IRESPred, and QGRS. ORF finder indicates four different potential translation initiations indicated by black arrows. In Position 6, an out of frame upstream AUG (uAUG) was identified. An in‐frame non‐AUG start potentially able to express an N‐terminal extended isoform is present in Position 101. Canonical M1‐AQP4 and M23‐AQP4 start sites are highlighted in Positions 128 and 194. RBPsite predicts three different PTBP1 binding sites and one binding site for HNRNPK/HNRPL. QGRS predicts G‐RNA quadruplexes (blue bi‐planar structures). Note that two G‐RNA quadruplexes in Positions 150–169 and 209–232 are just downstream of M1‐AQP4 and M23‐AQP4 AUG, respectively. Interestingly, the DDX17 binding site represented by the CACACCU sequence is present in positions 535–541. IRESPred indicates the presence of a potential IRES in Positions 132–280 that include two G‐RNA quadruplexes [Color figure can be viewed at wileyonlinelibrary.com]

We have analyzed the mouse M1‐AQP4 mRNA sequence searching for secondary structures, RBPs binding sites, IRES, G‐RNA quadruplexes, and alternative ORFs. To this aim, the reference sequence of mouse M1‐AQP4 mRNA was analyzed using free on‐line tools: ORF finder, mFOLD, RBPsite, IRESPred, and QGRS. Figure 5(a) reports the most stable secondary structure of mouse M1‐AQP4 mRNA as predicted by mFOLD (Δ*G* = −672.40 kcal/mol). The 5′UTR and M1‐AQP4 and M23‐AQP4 translation initiation signals are predicted in very stable stem‐loop structures (red box and magnified region in Figure 5(a)). This region was deeply analyzed by IRESPred to search for potential IRES. A potential IRES is predicted in Positions 132–280. As indicated in Figure 5(b), this IRES is localized downstream of the M1‐AQP4 AUG and included the M23‐AQP4 AUG. This indicates the potential contribution of this IRES structure to a cap‐independent M23‐AQP4 translation from M1‐AQP4 mRNA. Figure 5(b) reports data obtained in detail. In Positions 6 and 101, two different ORFs are found, an AUG‐start out‐of‐frame uORF and an in‐frame non‐AUG start uORF, respectively (first two black arrows from left to right in Figure 5(b)). Four different PTBP1 binding sites are identified in Positions 26, 36, 109, and 232 (yellow boxes in Figure 5(b)). HNRNPK and HRNPL1 binding sites are predicted in Positions 39–47 (green box in Figure 5(b)). Most interestingly, QGRS revealed three different potential G‐RNA quadruplexes in Positions 83–98, 150–169, and 209–232 (marked by red numbers), the latter two predicted downstream M1‐AQP4 and M23‐AQP4 AUG, respectively (black arrows). They are found to be close to PTBP1 and HNRNPs and are predicted as part of IRES that span the Positions 132–280. Recently, the RNA sequence CACACCU was identified as a binding site for DDX17 (Ngo et al., 2019). The mouse M1‐AQP4 mRNA contains exactly this sequence in two positions, 535 and 1,235 (blue boxes in Figure 5(b)).

## 4 DISCUSSION

Physiological and pathological stimuli actively change gene expression through finely regulated mechanisms. The Mouse *aqp4* gene expresses many AQP4 isoforms that control the AQP4 supramolecular assembly state and the AQP4 localization into microdomains of the astrocyte plasma membrane. How AQP4 expression is modulated in astrocytes remains still largely unknown.

To obtain new insights into AQP4 regulation mechanisms in the CNS, here we have used a mouse model in which the OAP‐forming M23‐AQP4 isoform has been deleted. We have already shown that the AQP4 expression is strongly affected in the brain of this animal model (de Bellis et al., 2020), suggesting a central role of M23‐AQP4 in maintaining AQP4 expression at physiological levels. Here, we have analyzed this model to explore how the regulation of AQP4 expression responds to the deletion of M23‐AQP4 in the spinal cord. In heterozygous mice, we found that M1‐AQP4 expression is directly correlated to M23‐AQP4 expression and more importantly that the M23/M1 ratio is similar in heterozygous and WT spinal cord. This suggests that some mechanism allows the balance between M1‐AQP4 and M23‐AQP4 expression. Starting from this evidence, we hypothesize that M23‐AQP4 controls M1‐AQP4 expression in order to maintain the AQP4‐isoforms ratio within a physiological range. To investigate this hypothesis, we have analyzed some crucial *aqp4* gene regulation mechanisms in WT and M23‐null spinal cord. In M23‐null mice, despite observing 95% downregulation of M1‐AQP4, M1‐AQP4 mRNA, splicing, and M1‐AQP4 protein degradation mechanisms were found to be unaffected. This led us to investigate the translational control as being potentially involved. To test this hypothesis, we performed an RNA‐protein pull down assay followed by quantitative MS to identify and quantify RBPs that differentially interact with M1‐AQP4 mRNA in the spinal cord of WT versus M23‐null mice. This approach has never been used before for AQP4. We found that the absence of M23‐AQP4 induces a regulation of the interaction between RBPs and M1‐AQP4 mRNA.

By RNA‐protein pulldown, we obtained direct evidence of the interaction between AQP4‐M1 mRNA and the cytoskeleton. Microtubule and tubulin‐associated proteins were found to be able to bind M1‐AQP4 mRNA and to be mainly unchanged between WT and M23‐null mice. Considering that ribonucleoprotein complexes are actively transported along microtubules and that microtubules and tubulin‐binding proteins contribute to this mechanism (Chudinova & Nadezhdina, 2018), we speculate that these M1‐AQP4 mRNA interacting proteins could contribute to the recently identified AQP4 mRNA translocation and local translation in astrocytes (Boulay et al., 2019). More importantly the pull‐down strategy allowed us to identify DDX17 and PTBP1 as new AQP4 RBPs. No data have been previously reported about the role of DDX17 in astrocytes and its role in AQP4 expression has not been described to date. DDX17 is a member of the large family of DEAD‐box RNA helicase proteins necessary for proper function of RNA in many cellular processes. DDX17 contributes to the regulation of gene expression at many levels, mRNA splicing, export, stability, localization, and translation (Cordin et al., 2006; Giraud et al., 2018; Gustafson & Wessel, 2010; Linder & Jankowsky, 2011; Xing et al., 2019). The role of DDX17 in the regulation of AQP4 expression and AQP4‐dependent water transport is validated here by RNAi in astrocyte primary culture. The data show that astrocytes express DDX17, that they express only M1‐AQP4 mRNA, that in agreement with our previous findings M23‐AQP4 protein is translated from M1‐AQP4 mRNA (Pisani, Rossi, et al., 2011) and that DDX17 is a negative regulator of AQP4 translation. In this respect, DDX17 knockdown upregulates AQP4 protein expression, leaving AQP4 mRNA levels unchanged, increases astrocyte swelling, but does not change PTBP1 (a positive regulator of AQP4 translation) expression. This strongly supports the direct role of DDX17 as a negative regulator of AQP4 translation.

It is important to note that DDX17 is the paralog of DDX5 and that DDX5 posttranscriptionally contributes to the regulation of myelin basic protein (MBP) levels in oligodendrocytes (Hoch‐Kraft et al., 2018). In particular, in line with data here obtained for DDX17 and AQP4, in oligodendrocyte, primary cultures DDX5 localizes in the nucleus and cytoplasm and its knockdown upregulates MBP leaving MBP mRNA unchanged, suggesting a role for DDX5 in the translational regulation of MBP. The exact mechanism by which DDX17 negatively controls AQP4 expression must be further characterized. As already suggested for DDX5 and MBP regulation (Hoch‐Kraft et al., 2018), DDX17 could be capable of remodeling AQP4 mRNA secondary structures, potentially interfering with translational activators or the association of silencing co‐factors.

Little is known about the role of DDX17 in the CNS. Only few papers report data about DDX17 in brain (Kircher et al., 2002; Luo et al., 2020; Moon et al., 2018) and no data are available about spinal cord. In one of these papers Ip et al. (2000) report that DEAD Box Protein p72 (the alternative name of DDX17) is downregulated in brain and muscle (two AQP4 expressing tissues) during development. Whether the DDX17 downregulation during brain development (Ip et al., 2000) contributes to the age‐dependent regulation of AQP4 expression in polarized astrocytes and blood brain barrier development (Gautam et al., 2020; Trillo‐Contreras et al., 2018) could be considered. This hypothesis is in line with the role of DDX5 in the MBP regulation and cell‐differentiation already reported for oligodendrocytes (Hoch‐Kraft et al., 2018).

By RNA‐protein pulldown, here we provide direct evidence that PTBP1 interacts with AQP4 mRNA in the spinal cord, that this interaction in M23‐null is greater than in WT mice and we show that PTBP1 is a positive regulator of AQP4 expression in astrocyte primary cultures. This evidence confirms data already obtained by luciferase‐assay in transfected HeLa cells by Baird et al. which showed the pivotal role of PTBP1 for M23‐AQP4 translation through an IRES‐dependent mechanism (Baird et al., 2007).We found that PTBP1 also controls the expression of M1‐AQP4 in astrocytes, despite being characterized as a translational regulator of M23‐protein translation (Baird et al., 2007). We speculate that astrocytes respond to M23‐AQP4 regulation by regulating M1‐AQP4 expression to maintain the physiological M1/M23 ratio.

Considering that in the spinal cord the M23‐AQP4 is five‐fold more abundant than M1‐AQP4 (Figure 1(c)) and that the elimination of M23‐AQP4 strongly also downregulates M1‐AQP4, the total AQP4 levels are extremely low in M23‐null mice. Starting from this evidence and from evidence obtained by RBP analysis, we hypothesize that in M23‐null spinal cord PTBP1 and DDX17 play a role in a mechanism aimed to compensate for the missing AQP4. This could explain why, compared to WT, in M23‐null mice PTBP1 is the most highly RBP and DDX17 is the lowest. It is possible to speculate that in M23‐null mice the almost total loss of AQP4 protein expression activates a positive translational feedback aimed at compensating for the absence of AQP4. In this mechanism the PTBP1 and DDX17 are regulated in the opposite manner to WT. In M23‐null mice the absence of the M23 AUG start codon completely and irreparably prevents M23‐AQP4 protein synthesis. This could contribute to maintaining the positive translational feedback mechanism continuously activated in M23‐null mice. The mechanistic scenario here proposed cannot be tested in AQ4 KO mice, because AQP4 KO mice express a small amount of truncated AQP4 mRNA (Ma et al. 1996). This highlights the unique opportunity provided by the M23‐null model here analyzed to investigate how AQP4 expression affects the interaction between AQP4 mRNA and RBP.

It is generally accepted that RNA molecules can fold into intricate shapes that can provide an additional layer of modulator that shapes posttranscriptional control of gene expression beyond that of their sequence. Among these, alternative ORFs), IRES, and G‐RNA quadruplexes play a key role in the translational regulation in a coordinated manner with RBPs (Leppek et al., 2018). Searching for these elements, here we show that mouse AQP4‐M1 mRNA contains RNA binding sites for DDX17 and PTBP1, supporting wet data obtained by RNA‐protein pull down. Furthermore, we show that the 5′ region of AQP4‐M1mRNA contains out‐of‐frame upstream ORF, in‐frame upstream ORF, G‐RNA quadruplexes, and one IRES. Despite the fact that all these predictions must be validated by wet‐experiments to confirm a role in the regulation of AQP4 expression, we suggest that the alteration of the binding with DDX17 and PTBP1 found here could play a synergic role with these structures to regulate AQP4 expression. A similar scenario has amply already been shown for other genes (Arora et al., 2008; Hansel‐Hertsch et al., 2017; Kumari et al., 2008; Leppek et al., 2018). The fact that spinal cord also expresses M23‐AQP4 mRNAs poses another level of control in which DDX17 and PTBP1 could play a role in the regulation of AQP4 expression. Whether and how DDX17 and PTBP1 regulate the M23 protein expression by modulating M23‐AQP4 mRNA structures must be further characterized.

In conclusion, the main novelty of this study is the characterization of M1‐AQP4 MBPs and the identification of the role of DDX17 in the regulation of AQP4 expression. This lays the foundation to understand more deeply how AQP4 is regulated in physiological and pathological conditions.

## CONFLICT OF INTEREST

The authors declare no conflict of interest.

## Supporting information

**Supplementary Figure 1** Amplification efficiency of AQP4‐tot and AQP4‐M1‐AQP4 specific primers using a single DNA template reveals that both pairs of primers have the same amplification efficiency and that the same Ct was observed for each input.

**Supplementary Figure 2** ΔCt between M1‐AQP4 mRNA and AQP4‐tot mRNA in spinal cord of WT and M23‐null mice. The AQP4‐M1‐AQP4 mRNA is not the only AQP4‐mRNA in spinal cord as indicated by one cycle of difference between total AQP4 mRNAs and M1‐AQP4 mRNA. n = 4 for each genotype.

**Supplementary Figure 3A:** PTBP1 western blotting analysis of RNA‐protein pull‐down eluates obtained using WT and M23‐null mice spinal cord extracts using PolyA‐coated and AQP4 mRNA‐coated beads. The PTBP1 interaction with AQP4 mRNA was double in M23‐null with respect to the WT mice and was absent in PolyA control coated beads (n = 2 for each genotype. Different animals were used from those used for the MS analysis shown in Figure 3(c). Student's *t* test for unpaired data). **B:** PTBP1 western blotting analysis of RNA‐protein pulldown eluates obtained using mouse spinal cord extracts with GAPDH‐coated and AQP4 mRNA‐coated beads. PTBP1 exclusively interacts with AQP4 mRNA ([n = 6], using different animals from those used for the MS analysis shown in Figure 3(c) and in Supplementary Figure 3(a)).

**Supplementary Figure 4** Western blotting analysis of PTBP1, GFAP and GAPDH expression in astrocyte primary culture treated with control and DDX17 siRNA, show that none of these proteins were changed by DDX17 knockdown (n = 3, Student's *t* test for unpaired data).

**TableS1**: Xxxx

## Data Availability

The mass spectrometry proteomics data have been deposited with the ProteomeXchange Consortium via the PRIDE partner repository with the dataset identifier PXD024792. The authors confirm that all other data supporting the findings of this study are available within the article.
