# Supplementary figures and images for: Regulation of aquaporin‐4 expression in the central nervous system investigated using M23‐AQP4 null mouse
Source: Glia. 2021 May 26;69(9):2235–51. doi: 10.1002/glia.24032 (PMC8361696; doi:10.1002/glia.24032)

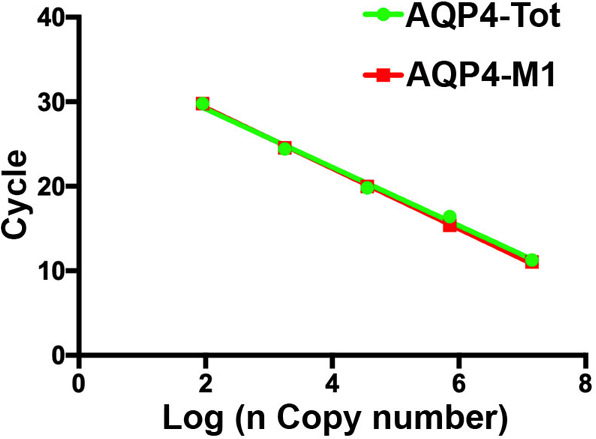

Supplement: Supplementary file 1 — Supplementary Figure 1 Amplification efficiency of AQP4‐tot and AQP4‐M1‐AQP4 specific primers using a single DNA template reveals that both pairs of primers have the same amplification efficiency and that the same Ct was observed for each input. [file GLIA-69-2235-s002.tif]

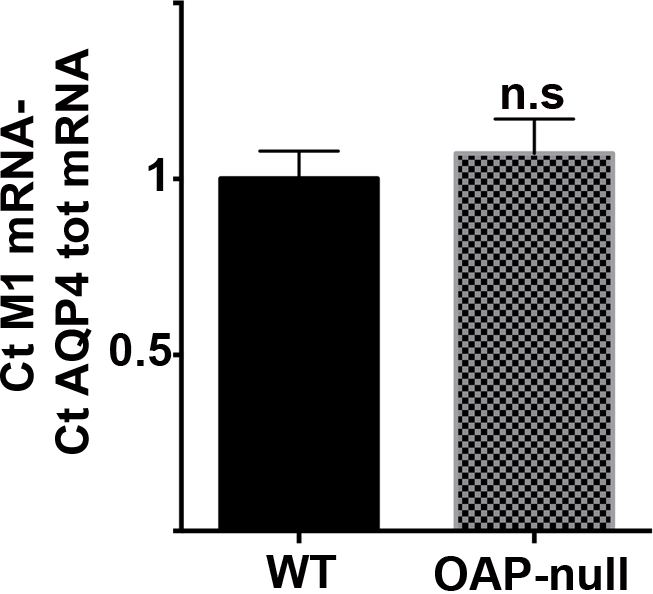

Supplement: Supplementary file 2 — Supplementary Figure 2 ΔCt between M1‐AQP4 mRNA and AQP4‐tot mRNA in spinal cord of WT and M23‐null mice. The AQP4‐M1‐AQP4 mRNA is not the only AQP4‐mRNA in spinal cord as indicated by one cycle of difference between total AQP4 mRNAs and M1‐AQP4 mRNA. n = 4 for each genotype. [file GLIA-69-2235-s003.tif]

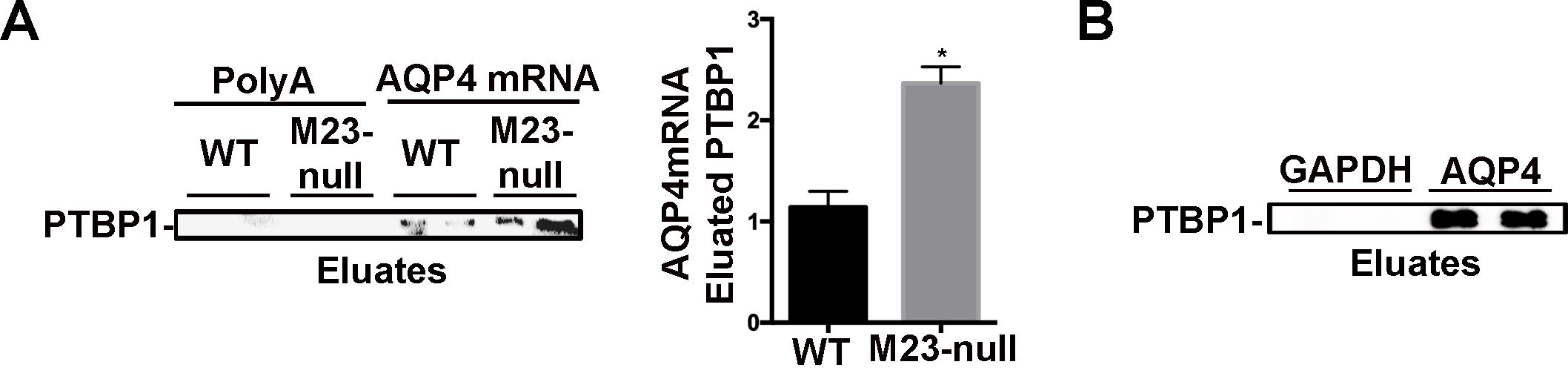

Supplement: Supplementary file 3 — Supplementary Figure 3A: PTBP1 western blotting analysis of RNA‐protein pull‐down eluates obtained using WT and M23‐null mice spinal cord extracts using PolyA‐coated and AQP4 mRNA‐coated beads. The PTBP1 interaction with AQP4 mRNA was double in M23‐null with respect to the WT mice and was absent in PolyA control coated beads (n = 2 for each genotype. Different animals were used from those used for the MS analysis shown in Figure 3(c). Student's t test for unpaired data). B: PTBP1 western blotting analysis of RNA‐protein pulldown eluates obtained using mouse spinal cord extracts with GAPDH‐coated and AQP4 mRNA‐coated beads. PTBP1 exclusively interacts with AQP4 mRNA ([n = 6], using different animals from those used for the MS analysis shown in Figure 3(c) and in Supplementary Figure 3(a)). [file GLIA-69-2235-s001.tif]

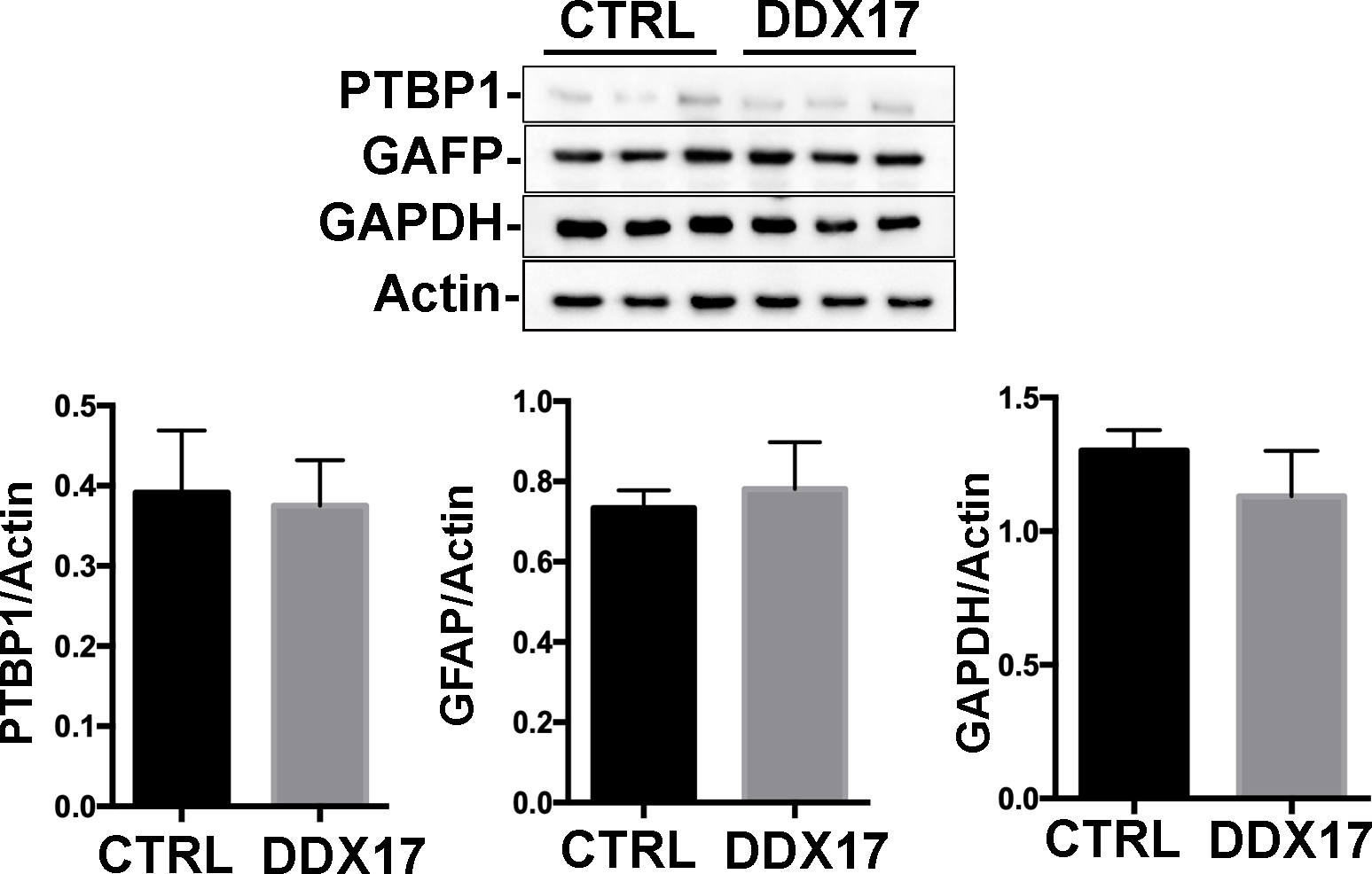

Supplement: Supplementary file 4 — Supplementary Figure 4 Western blotting analysis of PTBP1, GFAP and GAPDH expression in astrocyte primary culture treated with control and DDX17 siRNA, show that none of these proteins were changed by DDX17 knockdown (n = 3, Student's t test for unpaired data). [file GLIA-69-2235-s005.tif]
